# Protective Effects of Complement Component 8 Gamma Against Blood-Brain Barrier Breakdown

**DOI:** 10.3389/fphys.2021.671250

**Published:** 2021-06-03

**Authors:** Jong-Heon Kim, Jin Han, Kyoungho Suk

**Affiliations:** ^1^Brain Science and Engineering Institute, Kyungpook National University, Daegu, South Korea; ^2^Department of Pharmacology, School of Medicine, Kyungpook National University, Daegu, South Korea; ^3^Department of Biomedical Science, School of Medicine, Kyungpook National University, Daegu, South Korea

**Keywords:** complement component 8 gamma, sphingosine-1-phosphate, sphingosine-1-phosphate receptor 2, blood-brain barrier, astrocyte, neuroinflammation

## Abstract

The blood-brain barrier (BBB) regulates the traffic of micromolecules and macromolecules between the peripheral blood and the central nervous system, to maintain brain homeostasis. BBB disruption and dysfunction accompany a variety of neurological disorders and are closely related with the neuroinflammatory cascades that are triggered by leukocyte infiltration and glial activation. Here, we explored the role of complement component 8 gamma (C8G) in the maintenance of BBB integrity. Previously, C8G was shown to inhibit neuroinflammation by interfering with the sphingosine-1-phosphate (S1P)-S1PR2 interaction. The results of the present study revealed that C8G is localized in perivascular astrocytes, whereas S1PR2 is expressed in endothelial cells (ECs). In the lipopolysaccharide (LPS)-induced neuroinflammation model, the intracerebroventricular administration of the recombinant C8G protein protected the integrity of the BBB, whereas shRNA-mediated C8G knockdown enhanced BBB permeability and neutrophil infiltration. Using pharmacological agonists and antagonists of S1PR2, we demonstrated that C8G inhibited the inflammatory activation of ECs in culture by antagonizing S1PR2. In the *in vitro* BBB model, the addition of the recombinant C8G protein preserved endothelial integrity, whereas the knockdown of C8G exacerbated endothelial leakage under inflammatory conditions. Together, our findings indicate an important role for astrocytic C8G in protecting the BBB in the inflamed brain, suggesting a novel mechanism of cross talk between astrocytes and ECs in terms of BBB maintenance.

## Introduction

The blood-brain barrier (BBB), which comprises specialized brain microvascular endothelial cells (ECs), plays an important role in maintaining a flow of substances into and out of the brain. The BBB limits the passive diffusion of molecules into the brain *via* complex intercellular tight junctions between adjacent ECs. As a result, most molecular traffic is dependent on a selective transport system that shuttles required nutrients and metabolites into the brain and excludes potentially harmful compounds (Risau and Wolburg, 1990). Brain microvessels (capillaries) are surrounded by perivascular astrocytes. In turn, astrocytes form perivascular endfeet onto the abluminal side of brain capillaries and play a special role in the ionic, amino acid, neurotransmitter, and water homeostasis of the brain (Abbott et al., 2006). Given the anatomical relationships of the associated cells, synergistic intercommunication can be observed between two or more cell types. These signaling interactions may become particularly important in pathological conditions.

Blood-brain barrier integrity is critical for maintaining the microenvironment of the central nervous system. Numerous reports have demonstrated that disruption of the BBB is closely related with several neuropathologies, including brain injury (Chodobski et al., 2011), epilepsy (Marchi et al., 2012), and neurodegenerative diseases [e.g., Alzheimer’s disease (Sweeney et al., 2018), multiple sclerosis (Minagar and Alexander, 2003), and Parkinson’s disease (Gray and Woulfe, 2015)]. BBB disruption induces the extravasation of intravascular fluid and excessive infiltration of leukocytes into the brain parenchyma, causing neuroinflammation (Michinaga and Koyama, 2019). Thus, brain ECs stimulated by proinflammatory stimuli express leukocyte adhesion molecules and release cytokines. These events cause the alteration of tight junction proteins, leading to an increase in BBB permeability and the release of matrix metalloproteases. These events may trigger glial activation and neuroinflammation, ultimately causing brain damage (van Vliet et al., 2007; Zlokovic, 2008).

Recently, the role of sphingosine-1-phosphate (S1P) receptors (S1PRs) in the modulation of the endothelial BBB permeability was highlighted (Sanchez et al., 2003, 2007). S1PR1 and S1PR2, which are mainly expressed in ECs, are crucial for the proadhesion and proinflammatory phenotypes (Zhang et al., 2013). S1P is a multifunctional bioactive lipid mediator of cellular responses that exerts its functions by activating G-protein-coupled receptors, i.e., S1PRs. S1P is generated by the catabolism of sphingomyelin, which is an abundant component of biological membranes. Sphingomyelinase converts sphingomyelin to ceramide, which is further degraded to sphingosine by a ceramidase. In turn, sphingosine can be converted to S1P *via* the action of sphingosine kinase (SPHK) and ATP (Spiegel and Merrill, 1996; Canals et al., 2011). Under inflammatory conditions, the activity of SPHK1, which catalyzes the release of S1P, is increased in ECs (Tauseef et al., 2008; Fukuhara et al., 2012) and the interaction between S1P and S1PR2 leads to an increase in barrier permeability, proinflammatory molecules, and procoagulant molecules (Zhang et al., 2013; Kim et al., 2015). The activation of the Sphk1-S1P-S1PR2 axis is now known as a crucial cascade in the endothelial inflammatory response.

Our previous work demonstrated that the novel astrocytic complement component 8 gamma (C8G) is upregulated and acts as an antagonist of microglial S1PR2, resulting in the attenuation of neuroinflammation in both lipopolysaccharide (LPS)-injected and 5xFAD Alzheimer’s disease model mice (Kim et al., 2020). Interestingly, many astrocytes expressing C8G were detected in the perivascular region, as well as in the parenchyma. Based on our previous observations, we wondered whether astrocytic C8G suppresses acute endothelial inflammation by interrupting the activation of S1PR2. In this study, we explored the role of C8G in the acute inflammatory response of ECs and in BBB integrity. We characterized perivascular astrocytic C8G as a novel inhibitor of brain endothelial inflammation during neuroinflammation. Our findings emphasize the critical role of C8G in the endothelial responses to inflammation and highlight the potential utility of C8G as an S1PR2 inhibitor in the therapy of inflammatory disorders.

## Materials and Methods

### Reagents

Lipopolysaccharide from *Escherichia coli* 0111:B4 was purchased from Sigma-Aldrich (St. Louis, MO, United States). Recombinant mouse IFN-γ protein was purchased from R&D systems. The bacterially expressed recombinant mouse C8G protein was prepared as described previously (Lee et al., 2008). Briefly, the recombinant mouse C8G protein without the signal peptide was expressed in *E. coli* (strain BL21) as a glutathione S-transferase (GST) fusion protein. The protein was purified using glutathione Sepharose 4B beads (GE Healthcare, Chicago, IL, United States). GST was removed by thrombin digestion. C8G was purified and concentrated. SDS-PAGE with Coomassie Brilliant Blue R250 staining showed that the purity of C8G was over 95%. JTE013, S1P, STATTIC, and BAY 11-7082 were purchased from Cayman Chemical (Ann Arbor, MI, United States). CYM-5478 was purchased from Aobious Inc. (Gloucester, MA, United States).

### Mice

Male C57BL/6 mice (8–9 weeks of age) were purchased from Samtako Bio (Osan, South Korea). All mice were housed in groups of 3–5 animals per cage under specific pathogen-free conditions and a 12/12 h light/dark cycle. Neuroinflammation was induced by intraperitoneal (i.p.) injection of a vehicle (same volume of PBS) or LPS (5 mg/kg). When necessary, animals were sacrificed under CO_2_-induced anesthesia.

### Immunohistochemical Procedures

To stain microvessels, mice were anesthetized with ether and transcardially perfused with Cy3-conjugated tomato (*Lycopersicon esculentum*) lectin (100 μg of lectin per 100 μl of buffered saline; Vector Laboratories Inc., Burlingame, United States). Tomato lectin binds uniformly to the luminal surface of ECs (Thurston et al., 1996). After a 3-min period, the mice were perfused with 4% paraformaldehyde in PBS and their brains were extracted and postfixed and cryoprotected with a 30% sucrose solution for 3 days. For immunohistochemistry, mice were perfused with 4% paraformaldehyde in PBS under deep anesthesia. The fixed brains were then embedded in optimal cutting temperature compound (Tissue-Tek; Sakura Fine-Tek, Tokyo, Japan) and cut into 20-μm-thick sections on a cryostat. Brain sections were permeabilized in 0.1% Triton X-100 and blocked using 1% bovine serum albumin (BSA) and 5% normal donkey serum for 1 h at room temperature. Brain sections were incubated with the following primary antibodies at 4°C overnight: rabbit anti-C8G IgG (1:25; Cloud Clone Corp., Katy, TX, United States), rabbit anti-S1PR2 IgG (1:200; Proteintech Group Inc., Chicago, IL, United States), mouse anti-glial fibrillary acidic protein (GFAP) IgG (1:200; Novus Biologicals), and mouse anti-LY6G IgG (1:200, Thermo Scientific). This was followed by incubation for 2 h at room temperature with fluorescence-conjugated secondary antibodies: FITC-conjugated donkey anti-rabbit or anti-mouse IgG antibodies (1:200), Cy3-conjugated donkey anti-mouse IgG antibodies (1:200), or Cy5-conjugated donkey anti-rabbit IgG antibodies (1:200; Jackson ImmunoResearch Laboratories, West Grove, PA, United States). Finally, the sections were mounted and counterstained using DAPI-containing gelatin.

### Reverse Transcription-PCR

Total RNA was isolated from cells or whole brains using the QIAzol reagent (Qiagen, Hilden, Germany), according to the manufacturer’s instructions. Reverse transcription and PCR amplification were performed using a C1000 Touch thermal cycler (Bio-Rad, Hercules, CA, United States) using specific primer sets [*C8g*: forward (5'-CCCAGGTCAATTTCAGTGCT-3') and reverse (5'-GGTACAGGATGGCAAAGCTC-3'); *s1pr1*: forward (5'-CTCCACCGTGCTCCCGCTCTA-3') and reverse (5'-GGAGATGTTCTTGCGGAAGGTCAGG-3'); *s1pr2*: forward (5'-GCGTGGTCACCATCTTCTCC-3') and reverse (5'-CGTCTGAGGACCAGCAACATC-3'); *claudin5*: forward (5'-CCTTCCTGGACCACAACATC-3') and reverse (5'-GCCGGTCAAGGTAACAAAGA-3'); *occludin*: forward (5'-TTGGGACAGAGGCTATGG-3') and reverse (5'-ACCCACTCTTCAACATTGGG-3'); and *gapdh*: forward (5'-ATGGTGAAGGTCGGTGTG-3') and reverse (5'-ACCAGTGGATGCAGGGAT-3')]. *GAPDH* was used as a reference control. The nucleotide sequences of the primers were based on published cDNA sequences.

### Quantification of Evans Blue Extravasation

Evans blue extravasation was measured using a procedure described previously (Hyun et al., 2013). Evans blue dye (2% in saline, 4 ml/kg) was injected *via* the right femoral vein at 18 h after LPS injection (5 mg/kg) and was allowed to remain in circulation for 30 min. At the end of the experiments, the chest was opened, and mice were perfused transcardially with 250 ml of saline at a pressure of 110 mm Hg for approximately 10 min, until the fluid from the right atrium became colorless. Brain samples were weighed for the quantitative measurement of EB-albumin extravasation (Kaya et al., 2004). Brain samples were homogenized in 2.5 ml of phosphate-buffered saline and mixed by vortexing for 5 min after the addition of 2.5 ml of 60% trichloroacetic acid (TCA), to precipitate proteins. Samples were cooled and then centrifuged for 30 min at 1,000 × *g*. The supernatant was measured at 610 nm for absorbance of EB using a spectrophotometer. EB was expressed as μg/mg of brain tissue against a standard curve.

### Cell Culture

The mouse microvascular endothelial cell line bEnd.3 was purchased from the American Type Culture Collection. The media reagents that were used in this study were as follows: Dulbecco’s Modified Eagle’s Medium (DMEM), 10% fetal bovine serum (FBS), 1% penicillin, and streptomycin. The primary astrocyte culture was prepared from mixed glial cultures as described previously, with minor modifications (McCarthy and de Vellis, 1980). Briefly, whole brains from 3-day-old C57BL/6 mice were chopped and mechanically disrupted using a nylon mesh. The cells obtained were seeded in culture flasks and grown at 37°C in a 5% CO_2_ atmosphere in DMEM.

### Nitrite Quantification and Cell Viability Test

The NO_2_ secreted into the culture medium was measured to evaluate nitric oxide (NO) production in glial cells using the Griess reagent, as described previously (Lee and Suk, 2007). Cell viability was measured using the MTT assay, as described previously (Lee et al., 2009).

### Construction of rAAV Vectors Containing a Hybrid Adeno-Associated Virus Serotype (AAV-DJ) C8G shRNA

For *in vivo* gene silencing, the validated mouse shRNA sequence of C8G was cloned into the pSicoR vector using the HpaI/XhoI sites (Addgene, Cambridge, MA, United States), followed by subcloning into the pAAV-MCS vector (Stratagene, San Diego, CA, United States) using the MluI/BglII sites. High-titer rAAV vectors were produced in HEK293TN cells using a helper virus-free system. Briefly, the rAAV vectors were produced after co-transfection of equimolar amounts of a rep/cap/helper plasmid. After incubation for 72 h, the cells were lysed, treated with benzonase (Sigma-Aldrich), and further purified using HiTrap heparin columns (GE Healthcare). Amicon ultra-15 centrifugal filter units (Millipore, Billerica, MA, United States) were used to increase the concentration to the final volume.

### *In vitro* Permeability Assay

The bEnd.3 cells were grown on the inside of gelatin-coated transwell inserts (0.4 μm, Corning-Costar, Corning, NY, United States). Subsequently, 165 μg/ml of Evans blue/0.1% BSA (Sigma-Aldrich) were added to the upper chamber 1 h before measurements. The intensities of the diffused Evans blue/0.1% BSA in the lower chamber were measured at 650 and 485/535 nm, respectively. The results were calculated as the ratio of the Evans blue/0.1% BSA concentration in the lower chamber to the total concentration of Evans blue/0.1% BSA added to the upper chamber at the beginning of the experiment. Transendothelial electrical resistance (TEER) across the membrane was measured using an Ohm voltmeter (World Precision Instrument, Sarasota, FL, United States). The gelatin-coated transwell inserts were placed in 24-well plates containing culture medium and used to measure background resistance. The resistance measurements of these blank filters were subtracted from those of filters with cells. Values were measured as Ωcm^2^ based on the culture inserts.

### Statistical Analysis

Statistical analyses were performed using the Prism software, version 8.0 (GraphPad Software, La Jolla, CA, United States). Values are expressed herein as means ± SEM. Statistical analysis to compare the mean values among multiple groups was performed using a one-way ANOVA with correction for multiple comparisons. Comparison of two groups was performed using either Welch’s *t*-test or the unpaired nonparametric Mann-Whitney *U* test, based on identical interval and continuity, independence, normality, and equal variance. All experiments were repeated at least three times.

## Results

### C8G Expression in Perivascular Astrocytes of the Inflamed Brain

**Figure 1:**
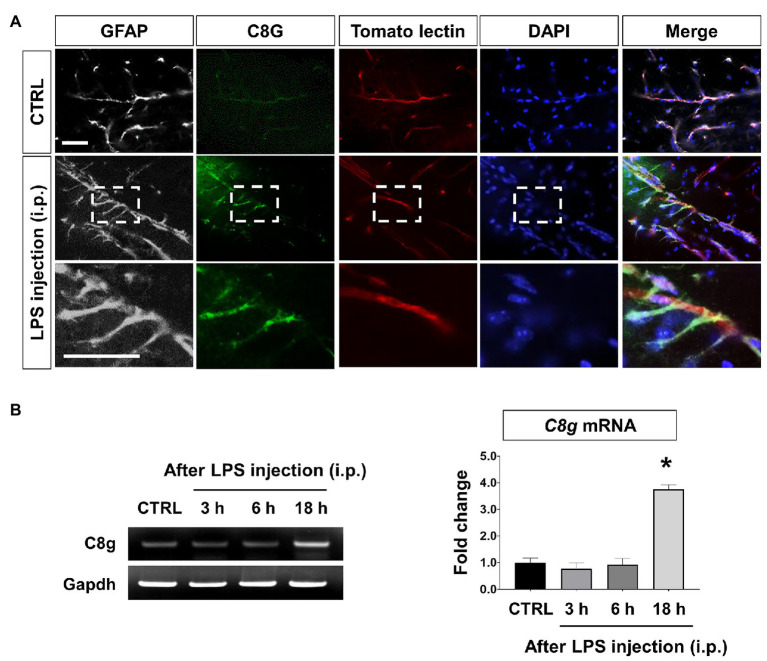
Component 8 gamma (C8G) expression in perivascular astrocytes in the lipopolysaccharide (LPS)-induced inflamed brain. **(A)** Immunofluorescence analysis of C8G in the hippocampus (green). An anti-GFAP antibody was used as a marker of astrocytes (gray). Tomato lectin stains brain blood vessels (red). Mice were injected with LPS (5 mg/kg) intraperitoneally (i.p.). Scale bar, 25 μm. **(B)** Time course of the C8G mRNA expression in the inflamed brain (*n* = 3). Data are the mean ± SEM. ^*^p < 0.05 vs. the control (CTRL), Dunnett’s multiple comparison *post hoc* test after one-way ANOVA.

Previously, we observed the astrocyte-specific induction of C8G in the inflamed brain (Kim et al., 2020). In this study, we detected the expression of C8G in perivascular astrocytes of the hippocampus of LPS-challenged mice (Figures 1A,B). These results led us to further explore whether astrocytic C8G plays a role in the modulation of the endothelium under inflammatory conditions. In the *in vitro* model, astrocytic C8G expression was remarkably induced by IL-1β or IL-6 and significantly reduced by BAY 11-7082 (NF-κB signaling inhibitor) or STATTIC (STAT3 signaling inhibitor; Supplementary Figure 1A). When we incubated primary astrocytes with LPS (1 μg/ml) for 6 h, C8G was slightly induced by LPS treatment; however, this difference was not significant (Supplementary Figure 1B). This implies that C8G expression is modulated by inflammatory mediators, rather than LPS. To confirm whether inflammatory mediators released from the endothelium could induce the expression of C8G in astrocytes, we examined the expression of C8G in astrocytes co-cultured with bEnd.3 ECs stimulated by LPS or LPS + IFN-γ for 24 h (Supplementary Figure 1C). The level of the C8G mRNA was significantly increased in the astrocytes that were co-cultured with the LPS + IFN-γ-stimulated bEnd.3 cells, but not in the astrocytes that were co-cultured with normal or the LPS-stimulated bEnd.3 cells. Although previous studies demonstrated that LPS can induce barrier disruption (Hu et al., 2019) and IL-6 production (Manni et al., 2011) in bEnd.3 cells, some reports revealed the low response of bEnd.3 cells to LPS-alone stimulation (Kacimi et al., 2011; Monnier et al., 2012). For example, Wang et al. (2011) also showed that LPS treatment significantly upregulated the expression of proinflammation cytokines, such as IL-1β, IL-6, and IL-8, in human umbilical vein endothelial cells (HUVECs), but not in human aortic endothelial cells (HAECs) and mouse microvascular ECs (bEND.3). Probably, LPS-alone treatment is not sufficient to induce a strong response and an inflammatory stimulation of bEnd.3 cells (Supplementary Figure 2). Together, our data indicates that astrocytic C8G expression can be induced by proinflammatory mediators released from ECs.

### C8G Reduces Evans Blue Extravasation and Neutrophil Infiltration *in vivo*

**Figure 2:**
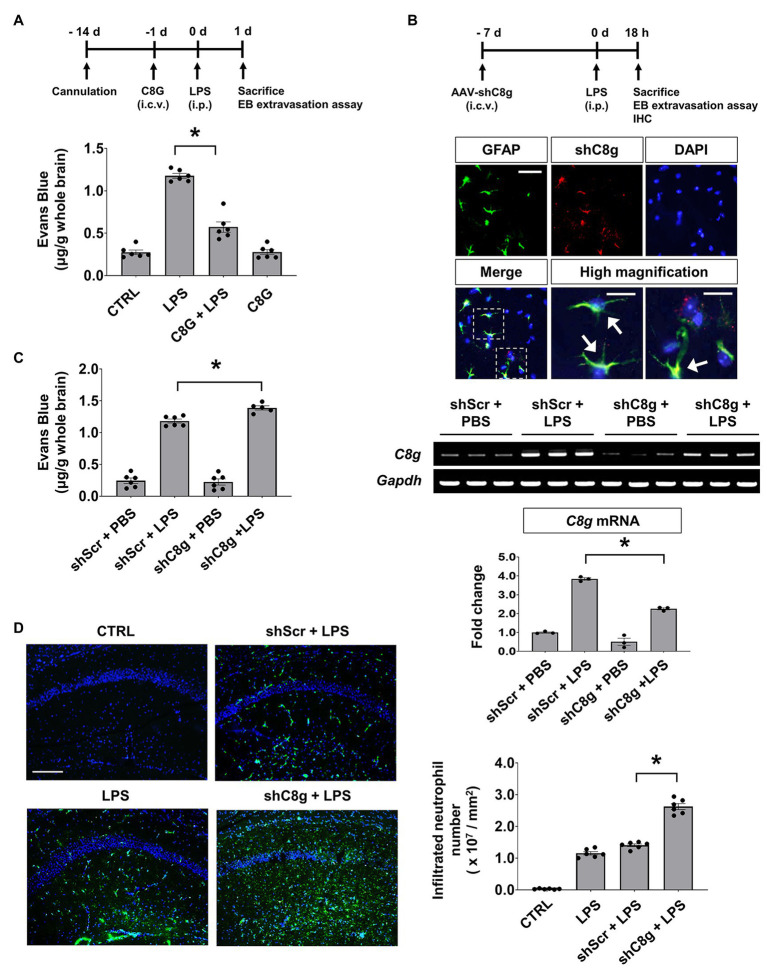
Complement component 8 gamma protects the blood-brain barrier (BBB) in the LPS-induced inflamed brain. **(A)** The administration of recombinant C8G protein inhibited Evans blue extravasation in the inflamed brain. Recombinant C8G protein (1 μg/ml) was injected intracerebroventricularly (i.c.v.) through a guide cannula before the intraperitoneal (i.p.) injection of LPS (5 mg/kg). C57BL/6 mice (*n* = 6 each) were sacrificed 24 h after the LPS challenge and their brains were removed. Brain vascular permeability was assessed by Evans blue accumulation in the brains, as described in the Materials and Methods. **(B)** Knockdown of C8G in the brain. Expression of adeno-associated virus (AAV)-shRNA C8g (shC8g, mCherry) in the hippocampus (scale bar, 25 μm) and high-magnification image of infected cells in the hippocampus (scale bar, 10 μm). Mice (*n* = 3) were given an i.c.v. injection of shC8g (1 × 10^9^ TU/ml, 2 μl) using a stereotaxic device and a microinjector. Brain tissues were stained with an anti-GFAP antibody (green) and DAPI (blue). The arrowheads indicate the co-localization of shC8g with GFAP. Knockdown efficacy was measured by reverse transcription-PCR (RT-PCR; 58.9% in the brain of LPS-injected mice, lower panel). **(C)** Knockdown of C8G increased Evans blue extravasation in the inflamed brain (*n* = 6 each). **(D)** Knockdown of C8G increased LPS-induced neutrophil infiltration. Infiltrated neutrophils in the hippocampus were immunostained with an anti-LY6G antibody (green) 18 h after LPS (5 mg/kg) i.p. injection (*n* = 6 each). Scale bar, 200 μm. All data are the mean ± SEM. ^*^p < 0.05, Student’s *t*-test **(C)** or Tukey’s multiple comparisons test after one-way ANOVA **(A,B,D)**.

To examine the role of C8G in the behavior of the endothelium *in vivo* under inflammatory conditions, we determined whether C8G could modulate BBB integrity using a recombinant C8G protein and shRNA-mediated knockdown of C8G. For this, Evans blue dye extravasation and neutrophil infiltration were assessed after LPS injection (i.p.). The results of this experiment revealed that Evans blue dye leakage was clearly increased at 24 h after LPS injection. However, recombinant C8G protein injection (i.c.v.) significantly reduced the Evans blue leakage (Figure 2A, ^*^p < 0.05). In turn, the knockdown of C8G increased the extravasation of the dye (Figures 2B,C, ^*^p < 0.05) and neutrophil infiltration (Figure 2D, ^*^p < 0.05). These results imply that C8G plays a potential role in the modulation of BBB integrity.

### C8G Inhibits Endothelial Activation by Blocking S1PR2 Activation

**Figure 3:**
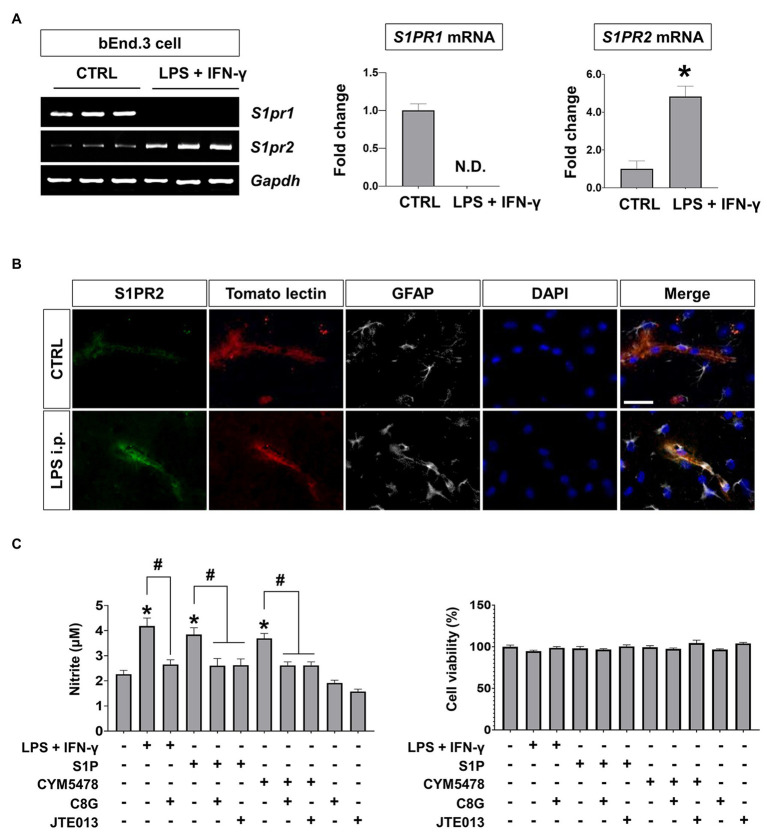
S1PR2 induction and inhibitory effect of C8G on nitric oxide production in endothelial cells (ECs) under inflammatory conditions. **(A)** S1PR1 and S1PR2 expression in ECs after inflammatory stimulation. bEnd.3 cells were stimulated with LPS (1 μg/ml) and IFN-γ (50 U/ml) for 6 h, followed by the extraction of total RNA. Alterations in S1PR1 and S1PR2 mRNA expression were analyzed by RT-PCR (*n* = 3). CTRL, control. **(B)** Immunofluorescence analysis of S1PR2 in the hippocampus. Mice were injected with LPS (5 mg/kg) intraperitoneally (i.p.). Tomato lectin stains brain blood vessels (red). An anti-GFAP antibody was used as a marker of astrocytes (gray). Scale bar, 25 μm. **(C)** C8G inhibited nitric oxide production in bEnd.3 cells. bEnd.3 cells were stimulated with LPS (1 μg/ml) + IFN-γ (50 U/ml), sphingosine-1-phosphate (S1P; 1 μM), or CYM5478 (1 μM) for 24 h after a 2 h pretreatment with C8G (1 μg/ml) or JTE013 (1 μM; *n* = 3). Data are the mean ± SEM. ^*^p < 0.05 vs. the control, ^#^*p* < 0.05, Student’s *t*-test **(A)** or Tukey’s multiple comparisons test after one-way ANOVA **(C)**.

Because we detected an interaction between C8G and S1PR2 in our previous work, we confirmed the expression of S1PR1 and S1PR2, which have been reported as major S1PR subtypes in ECs, in the endothelium under inflammatory conditions. The results of this experiment revealed that S1PR1 was greatly downregulated and S1PR2 was remarkably upregulated by inflammatory stimulation (LPS + IFN-γ) in the bEnd.3 mouse brain microvascular cell line (Figures 3A,B, ^*^p < 0.05). These data indicate that the role of S1PR2 may be predominant in the endothelium under inflammatory conditions. Consistently, it has been reported that S1PR2 activation in the endothelium is critical in acute vascular inflammation and loss of expression of tight junction genes (Zhang et al., 2013). Next, we examined the potential role of C8G in the acute inflammatory activation of ECs and compared the effects of a pharmacological agonist or antagonist on S1PR2. We measured the level of endothelial NO, which is critical for vascular leakage in acute endothelial cell activation (Bucci et al., 2005), after the application of the recombinant C8G protein or JTE013 (S1PR2 antagonist) to bEnd.3 cells stimulated by LPS + IFN-γ, S1P, or CYM5478 (S1PR2 agonist). The resulting data indicated a significant inhibitory effect of C8G on NO production in bEnd.3 cells stimulated by LPS + IFN-γ or the S1PR2 agonist (Figure 3C, ^#^*p* < 0.05). The inhibitory effect of C8G was similar to that of JTE013. These data suggest that C8G is an inhibitor of endothelial S1PR2 activation.

### C8G Rescues the Loss of Endothelial Tightness *in vitro*

**Figure 4:**
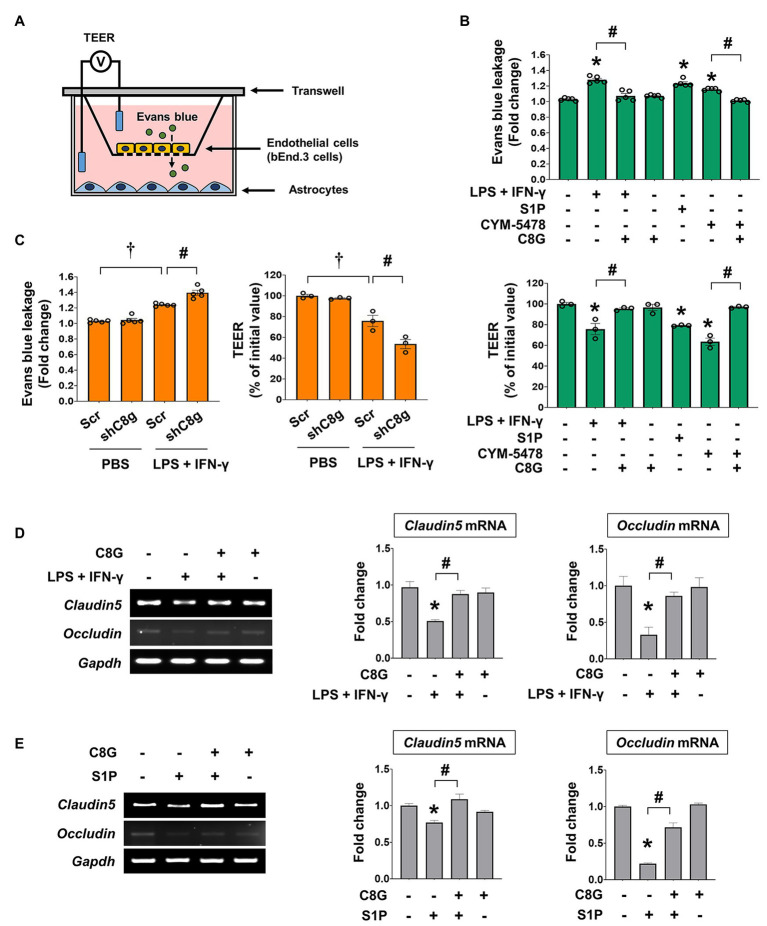
Complement component 8 gamma modulates the permeability of ECs after inflammatory stimulation. **(A)** Schematic representation of the *in vitro* model of the BBB. **(B)** C8G attenuated the permeability of ECs after inflammatory stimulation. bEnd.3 cells were grown on the inside of transwell inserts (apical side), and mouse primary astrocytes were cultured on the bottom side (basolateral side). Cells were pretreated with recombinant C8G (1 μg/ml) for 2 h and then exposed to LPS (1 μg/ml) + IFN-γ (50 U/ml), S1P (1 μM), or CYM5478 (1 μM) for 24 h. Left panel: Evans blue leakage was measured as described in the Materials and Methods. Data are presented as fold change compared with the control. Right panel: the transendothelial electrical resistance (TEER) across the membrane was assessed after pretreatment of the C8G protein and the administration of LPS and IFN-γ, S1P, or CYM5478. **(C)** Knockdown of C8G exacerbated the permeability of ECs after inflammatory stimulation. bEnd.3 cells were grown on the inside of transwell inserts (apical side), and mouse primary astrocytes infected by AAV-shRNA C8g were cultured on the bottom side (basolateral side). After the administration of the treatments described in **(B)**, Evans blue leakage (left panel) and TEER (right panel) were evaluated. **(D)** The recombinant C8G protein rescued the mRNA level of tight junction genes, which were downregulated by LPS + IFN-γ in bEnd.3 cells. After a 2 h C8G pretreatment (1 μg/ml), bEnd.3 cells were exposed to LPS (1 μg/ml) and IFN-γ (50 U/ml) for 12 h (*n* = 3 each). **(E)** The recombinant C8G protein rescued the mRNA level of tight junction genes, which were downregulated by S1P in bEnd.3 cells. After a 2 h C8G pretreatment (1 μg/ml), bEnd.3 cells were exposed to S1P (1 μM) for 12 h (*n* = 3 each). Data are the mean ± SEM. ^*^p < 0.05 vs. the control, ^#^*p* < 0.05, and ^†^*p* < 0.05 vs. shScr + PBS; Tukey’s multiple comparisons test after one-way ANOVA.

Next, we tested whether C8G could rescue the loss of endothelial integrity induced by inflammatory stimulation. For this, we utilized the *in vitro* BBB model that uses transwell plates (a non-contact co-culture system; Helms et al., 2016), which allows intercellular communication between ECs and astrocytes *via* diffusible factors, such as cytokines and C8G (Figure 4A). Using this model, we added inflammatory stimuli (LPS + IFN-γ), S1P, or CYM-5478 (S1PR2 agonist) to the upper wells, which contained cultures of bEnd.3 cells. The treatments significantly reduced tightness, as reflected by the increase of Evans blue dye penetration and a lower TEER value (Figure 4B, ^*^p < 0.05). However, the pretreatment of recombinant C8G with LPS + IFN-γ, S1P, or CYM-5478 enhanced endothelial tightness (Figure 4B, ^#^*p* < 0.05). To examine the direct role of C8G in this process, we confirmed the effect of C8G knockdown in this model (Figure 4C). Our data revealed that the knockdown of C8G significantly aggravated endothelial leakage (^#^*p* < 0.05). In addition, the decrease in the mRNA expression of tight junction genes (claudin5 and occludin) after LPS + IFN-γ or S1P treatment (Figures 4D,E, ^*^p < 0.05) was significantly alleviated by the pretreatment of the recombinant C8G protein (Figures 4D,E, ^#^*p* < 0.05). These data proved that C8G protects the BBB *in vitro*.

## Discussion

### C8G Is Induced During Acute Inflammatory Conditions

Complement component 8 gamma has been identified as one of the three subunits that constitute the complement component 8, which participates in the formation of the membrane attack complex on bacterial cell membranes. In our previous study, we uncovered an unexpected expression pattern and novel role of astrocytic C8G in the brain (Kim et al., 2020). That study showed the astrocyte-specific upregulation of C8G, independent of other subunits. In the present study, we found that C8G was also upregulated in the perivascular astrocytes in the inflamed brain (Figure 1A). C8G induction can be stimulated by inflammatory stimuli, mainly IL-1β and IL-6, which are acute-phase-response reactants in inflammatory conditions. In fact, the present data revealed that the pharmacological inhibitors BAY 11-7082 (NF-κB inhibitor) or STATTIC (STAT3 inhibitor), which inhibit the IL-6 or IL-1β signaling axes, respectively, blocked C8G induction in astrocytes significantly (Supplementary Figure 1A). Therefore, C8G expression in perivascular astrocytes may be induced by inflammatory mediators released from ECs, to protect the brain against cerebral vascular inflammation.

### C8G Protects the Blood-Brain Barrier

**Figure 5:**
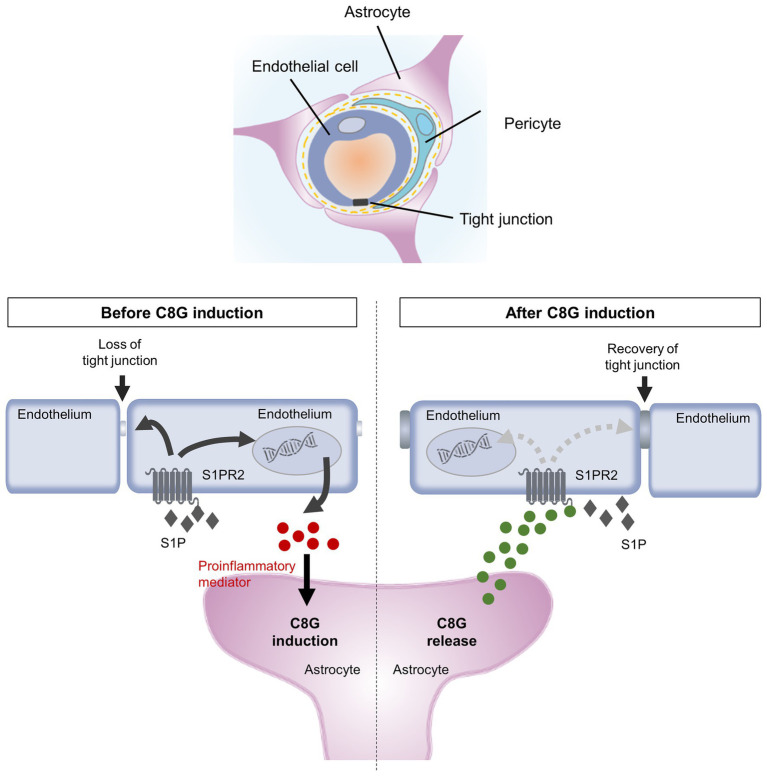
Schematic diagram summarizing our findings. Inflammatory stimuli activate SPHK-1, thus facilitating S1P release (Xia et al., 1998; Tauseef et al., 2008; Fukuhara et al., 2012) and upregulating proinflammatory mediators in ECs. Endothelial S1PR2 expression is upregulated and activated by extracellular S1P, which induces the expression of proinflammatory and procoagulant molecules and reduces endothelial integrity (Zhang et al., 2013). Moreover, proinflammatory mediators, such as IL-1β and IL-6, promote C8G induction in perivascular astrocytes. The C8G released from astrocytes interacts with endothelial S1PR2, resulting in blockage of inflammatory activation and BBB dysfunction. The intercommunication between astrocytes and ECs plays a crucial role in the modulation of the integrity of the BBB.

Our previous work demonstrated that C8G modulates microglial activation *via* the inhibition of S1PR2 activity during neuroinflammation (Kim et al., 2020). C8G inhibits the LPS-induced activation of the microglial RhoA-ROCK-NF-kB axis *via* competitive inhibition of the binding of S1P to S1PR2. In the present study, we found that perivascular astrocytic C8G modulated BBB permeability by interrupting endothelial S1PR2 activation. S1PRs, which were originally named endothelial differentiation gene receptors (Lee et al., 1998), are abundant in the endothelium and modulate BBB behaviors, including cell proliferation and survival and tight junction assembly, migration, and maturation (Hla et al., 2001; Spiegel and Milstien, 2003). Therefore, we assumed that the inhibitory role of C8G in S1PR2 activation may have an effect on BBB behavior. In fact, the Sphk activity can be rapidly increased by various extracellular stimuli, such as the platelet-derived growth factor (Olivera and Spiegel, 1993), tumor necrosis factor (Xia et al., 1998, 1999), vascular endothelial cell growth factor (Shu et al., 2002), epidermal growth factor (Meyer zu Heringdorf et al., 1999), and estrogen (Sukocheva et al., 2003); furthermore, the S1P-S1PR2-G12/13-Rho-ROCK-PTEN pathway increases BBB permeability and antagonizes the protective S1PR1 activation (Sanchez et al., 2005, 2007). In our *in vivo* study, C8G attenuated Evans blue extravasation, indicating BBB disruption, and knockdown of C8G increased the extravasation of this dye, as well as the infiltration of neutrophils in the brains of an LPS-induced neuroinflammation mouse model (Figure 2). Moreover, C8G inhibited endothelial cell activation (Figure 3), and endothelial permeability was decreased by recombinant C8G treatment and by knockdown of C8G in our *in vitro* BBB model (Figure 4). These findings argue in favor of an additional protective role of C8G during BBB disruption and neuroinflammation (Figure 5).

### Bidirectional Astroglial-Endothelial Induction Is Necessary to Maintain the Blood-Brain Barrier

The BBB is formed by capillary ECs, which are surrounded by the basal lamina and astrocytic perivascular endfeet. The basal lamina provides mechanical support for cell attachment, can be a cellular matrix, serves as a substratum for cell migration, separates adjacent tissue, and can act as a barrier to the passage of macromolecules (Thomsen et al., 2017). Astrocytes derived from ependymoglia of the developing neural tube retain some features of their original apical-basal polarity and have a more specific polarization function in relation to particular cell-cell associations in the adult brain (Abbott et al., 2006). Accumulating evidence demonstrates that astrocytes can promote many BBB features, such as the induction of tighter tight junctions (Dehouck et al., 1990; Rubin et al., 1991), expression and polarized localization of transporters, and specialized enzymatic systems (Abbott, 2002; Abbott et al., 2006). Considering the anatomical relationships of the associated cells, it is not surprising to find synergistic intercommunication involving more than one cell type. Various astrocyte-derived mediators, many of which have their own receptors in the brain endothelium, could be orchestrated by a variety of distinct and complex responses of brain ECs (Rubin et al., 1991). Conversely, the brain endothelium can enhance the growth and differentiation of astrocytes (Estrada et al., 1990; Mizuguchi et al., 1997; Schroeter et al., 1999).

Under pathological conditions, the endothelium allows the induction of endothelial receptors, which are normally suppressed, to form a new intercellular communication between the endothelium and adjacent cells, such as astrocytes. For example, perivascular astrocytes release vascular permeability factors, such as vascular endothelial growth factors, resulting in endothelial apoptosis and downregulation of tight junction proteins by interacting with their receptors in ECs (Jiang et al., 2014). In some cases, astrocyte-derived factors, such as the transforming growth factor beta, glial cell line-derived neurotrophic factor, and basic fibroblast growth factor, can promote BBB integrity by interacting with endothelial receptors (Leybaert et al., 1998; Michinaga and Koyama, 2019; Rui et al., 2019), to repair the barrier (Abbott et al., 1992, 2006). In turn, the leukemia inhibitory factor from brain ECs can induce astrocytic differentiation (Mi et al., 2001). The present study demonstrated that cytokines, particularly IL-1β and IL-6, released from the brain endothelium or other cells promote C8G induction in astrocytes, and that C8G diffused from astrocytes inhibits S1PR2 activity, causing acute endothelial inflammation and increasing BBB permeability. Therefore, our findings intimate a novel astrocyte-endothelium interaction.

We have demonstrated the existence of specific interactions between the brain endothelium and astrocytes under acute inflammatory conditions. This mutual induction was associated with the regulation of acute endothelial inflammation and BBB protection. Several neurological disorders exhibit an early BBB dysfunction. Recently, BBB leakage was suggested as a diagnostic feature of early Alzheimer’s disease (van de Haar et al., 2016; Nation et al., 2019). Therefore, early intervention for BBB protection may reduce the severity of neuropathological symptoms and facilitate recovery. For this, a better understanding of the mechanisms involved in the endothelial-astrocytic interaction is required. Therefore, our finding of an interaction between astrocytic C8G and endothelial S1PR2 may help the design of therapies based on early protection and repair of the BBB.

## Data Availability Statement

The original contributions presented in the study are included in the article/Supplementary Material, further inquiries can be directed to the corresponding authors.

## Ethics Statement

The animal study was reviewed and approved by Kyungpook National University Animal Care Committee (KNU 2015-0010).

## Author Contributions

JHK, JH, and KS designed the research. JHK and JH conducted experiments and acquired the data. JHK and KS drafted the manuscript, with final editing by all the authors and supervised the work. All authors contributed to the article and approved the submitted version.

### Conflict of Interest

The authors declare that the research was conducted in the absence of any commercial or financial relationships that could be construed as a potential conflict of interest.
