# Supplementary material for: Protective Effects of Complement Component 8 Gamma Against Blood-Brain Barrier Breakdown
Source: Front Physiol. 2021 Jun 3;12:671250. doi: 10.3389/fphys.2021.671250 (PMC8209513; doi:10.3389/fphys.2021.671250)
Supplement: Supplementary file 1 [file Data_Sheet_1.pdf]

frontiers

Supplementary Material

Protective effects of complement component 8 gamma against blood–brain barrier breakdown

Jong-Heon Kim[1*], Jin Han[2], Kyoungho Suk[1,2*]

[1]Brain Science and Engineering Institute, Kyungpook National University, Daegu, Republic of Korea.

[2]Department of Pharmacology and Department of Biomedical Science, School of Medicine, Kyungpook National University, Daegu, Republic of Korea.

*** Correspondence:**

Jong-Heon Kim jongheonkim@knu.ac.kr

Kyoungho Suk ksuk@knu.ac.kr

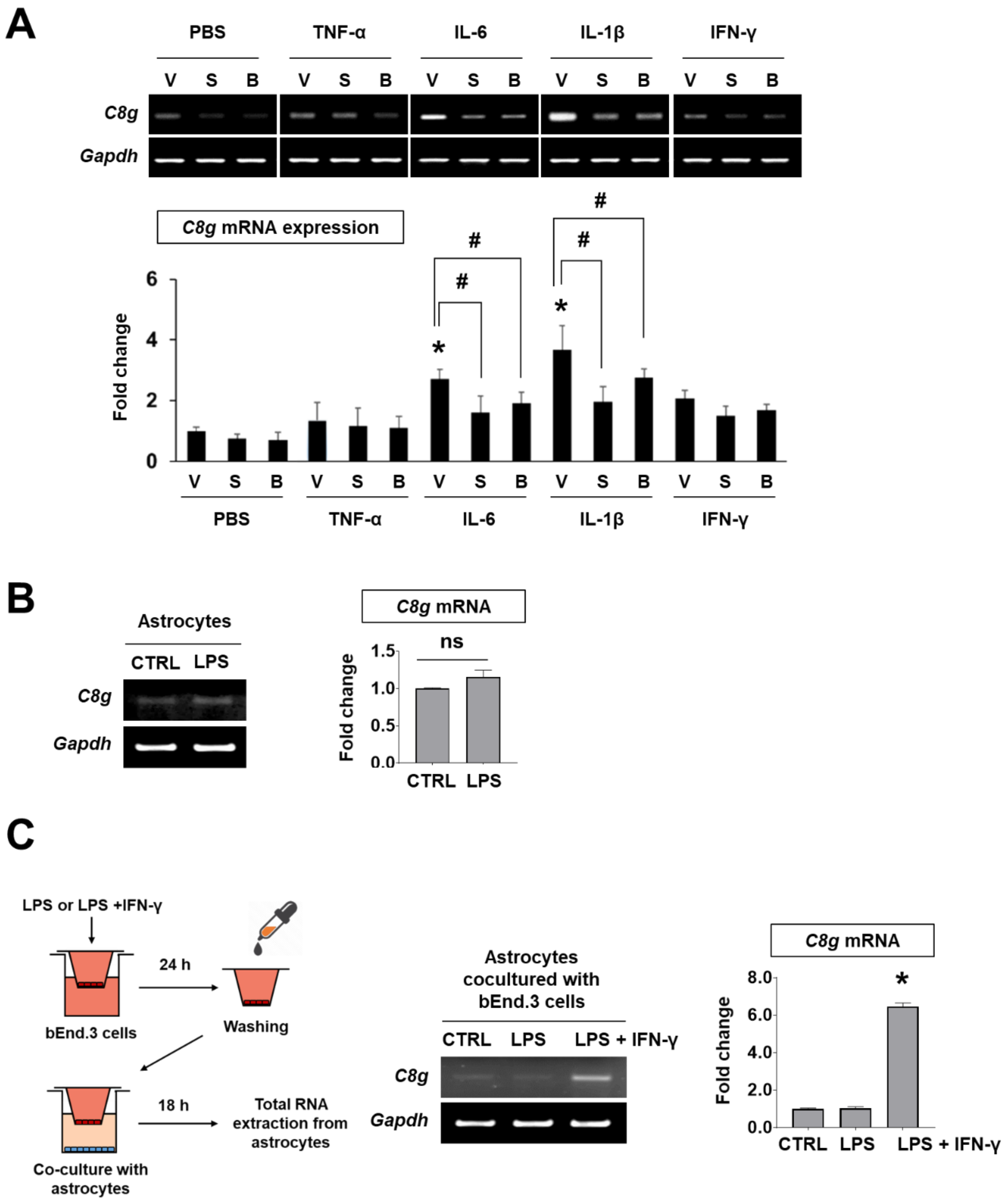

Supplementary Figure 1. Proinflammatory mediators modulate C8G induction. (A) Primary cultured astrocytes were applied to TNF-α (10 ng/ml), IL-1β (10 ng/ml), IL-6 (10 ng/ml), or IFN-γ (50 U/ml) with vehicle (V), STATTIC (10 μM, STAT3 inhibitor, S), or BAY 11-7082 (10 μM, NF-κB inhibitor, B) for 6 h (n = 3 each). Data are the mean ± SEM. $^{*}P < 0.05$ vs. the control (PBS + Vehicle), $^{\#}P < 0.05$, Tukey's multiple comparisons test after one-way ANOVA. (B) Induction of C8G in the astrocytes stimulated by LPS. Primary cultured astrocytes were applied to LPS (1 μg/ml) and incubated for 6 h (n = 3 each). Data are the mean ± SEM. ns, not significant. Student's *t*-test. (C) Induction of C8G in the astrocytes cocultured with bEnd.3 endothelial cells stimulated by LPS (1 μg/ml) or LPS (1 μg/ml) + IFN-γ (50 U/ml) (n = 3 each). Data are the mean ± SEM. $^{*}P < 0.05$ vs. the control (CTRL). Dunnett's multiple comparison post-hoc test after one-way ANOVA.

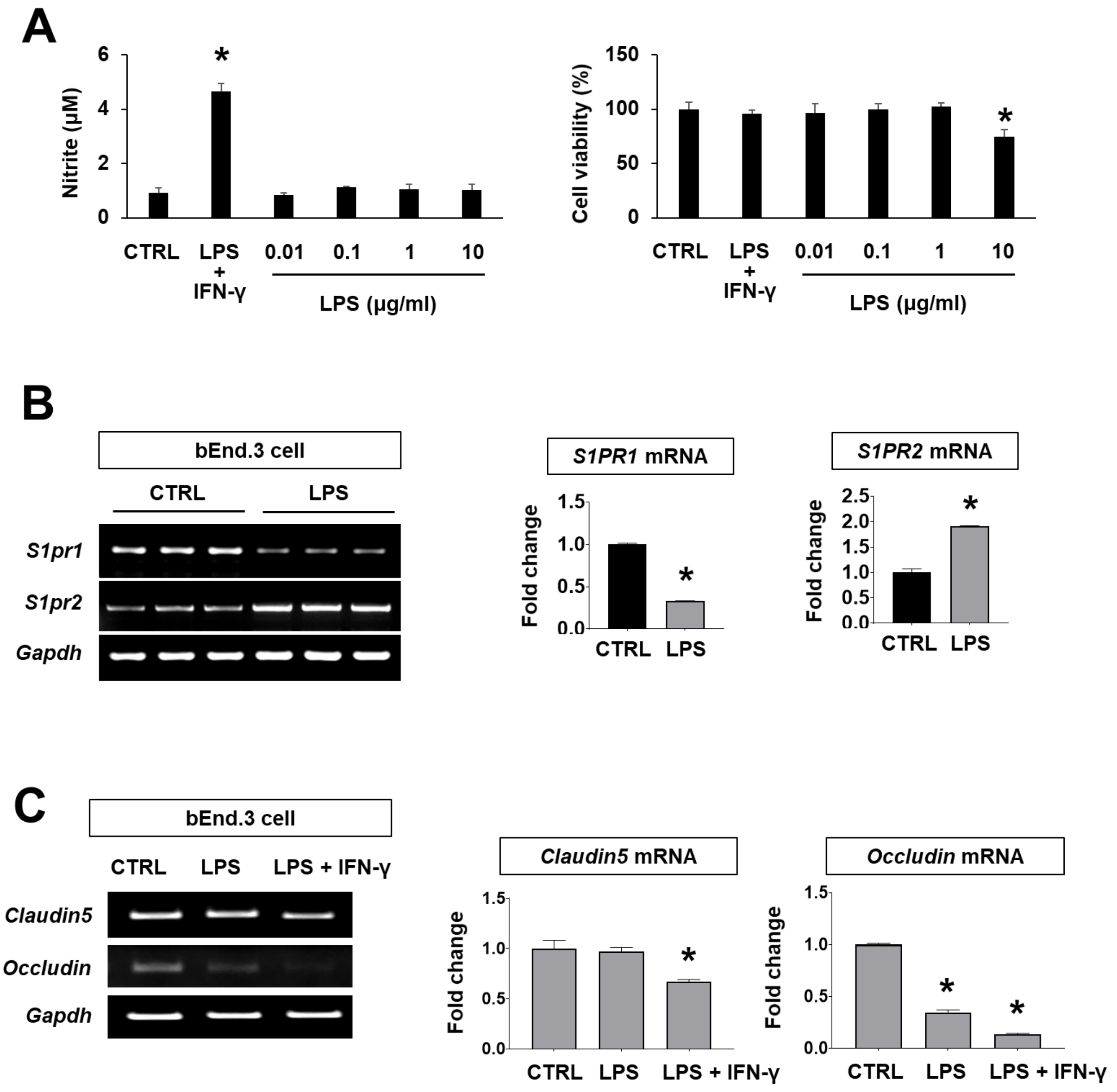

Supplementary Figure 2. Cellular responses of bEnd.3 cells stimulated by LPS-alone treatment. (A) Nitric oxide production and cell viability by LPS in bEnd.3 cells. Cells were stimulated with LPS (1 μg/ml) + IFN-γ (50 U/ml) or LPS as indicated. (B) S1PR1 and 2 expression in ECs after LPS stimulation. bEnd.3 cells were stimulated with LPS (1 μg/ml) for 6 h, followed by the extraction of total RNA. Alterations in S1PR1 and 2 mRNA expression were analyzed by RT-PCR (n = 3). (C) Claudin5 and occludin expression in ECs after LPS treatment. bEnd.3 cells were exposed to LPS (1 μg/ml) and LPS (1 μg/ml) + IFN-γ (50 U/ml) for 12 h (n = 3 each). Data are the mean ± SEM. $^{*}P < 0.05$ vs. CTRL, control. Tukey's multiple comparisons test after one-way ANOVA (A and C) and Student's *t*-test (B).
